# The use of antibiotics in the intensive care unit of a tertiary hospital in Malawi

**DOI:** 10.1186/s12879-020-05505-6

**Published:** 2020-10-19

**Authors:** Raphael Kazidule Kayambankadzanja, Moses Lihaka, Andreas Barratt-Due, Mtisunge Kachingwe, Wezzie Kumwenda, Rebecca Lester, Sithembile Bilima, Jaran Eriksen, Tim Baker

**Affiliations:** 1 Department of Anaesthesia and Intensive Care, Queen Elizabeth Central Hospital, Blantyre, Malawi; 2 College of Medicine, University of Malawi, Blantyre, Malawi; 3 Department of Emergencies and Critical Care, Oslo University Hospital, Oslo, Norway; 4 Liverpool School of Tropical Medicine, Liverpool, UK; 5 Malawi Liverpool Wellcome Trust Clinical Research Programme, Blantyre, Malawi; 6 Department of Global Public Health, Karolinska Institutet, Stockholm, Sweden; 7 Department of Infectious Diseases/Venhälsan, Stockholm South Hospital, Stockholm, Sweden; 8 Perioperative Medicine and Intensive Care, Karolinska University Hospital, Stockholm, Sweden

**Keywords:** Antibiotics, Antibiotic resistance, ICU, Malawi, Africa

## Abstract

**Background:**

Antibiotic resistance is on the rise. A contributing factor to antibiotic resistance is the misuse of antibiotics in hospitals. The current use of antibiotics in ICUs in Malawi is not well documented and there are no national guidelines for the use of antibiotics in ICUs. The aim of the study was to describe the use of antibiotics in a Malawian ICU.

**Methods:**

A retrospective review of medical records of all admissions to the main ICU in Queen Elizabeth Central Hospital in Blantyre, Malawi, between January 2017 and April 2019. Data were extracted from the ICU patient register on clinical parameters on admission, diagnoses, demographics and antibiotics both prescribed and given for all patients admitted to the ICU. Usage of antibiotics in the ICU and bacterial culture results from samples taken in the ICU and in the peri-ICU period, (from 5 days before ICU admission to 5 days after ICU discharge), were described.

**Results:**

Six hundred-and-forty patients had data available on prescribed and received medications and were included in the analyses. Of these, 577 (90.2%) were prescribed, and 522 (81.6%) received an antibiotic in ICU. The most commonly used antibiotics were ceftriaxone, given to 470 (73.4%) of the patients and metronidazole to 354 (55.3%). Three-hundred-and-thirty-three (52.0%) of the patients received more than one type of antibiotic concurrently – ceftriaxone and metronidazole was the most common combination, given to 317 patients. Forty five patients (7.0%) were given different antibiotics sequentially. One-hundred-and-thirty-seven patients (21.4%) had a blood culture done in the peri-ICU period, of which 70 (11.0% of the patients) were done in the ICU. Twenty-five (18.3%) of the peri-ICU cultures were positive and eleven different types of bacteria were grown in the cultures, of which 17.2% were sensitive to ceftriaxone.

**Conclusion:**

We have found a substantial usage of antibiotics in an ICU in Malawi. Ceftriaxone, the last-line antibiotic in the national treatment guidelines, is commonly used, and bacteria appear to show high levels of resistance to it, although blood culture testing is infrequently used. Structured antibiotic stewardship programs may be useful in all ICUs.

**Supplementary information:**

The online version contains supplementary material available at 10.1186/s12879-020-05505-6.

## Introduction

Antibiotic resistance is on the rise. Globally, the estimated 700,000 people who die each year due to drug resistant infections is projected to increase to 10 million a year by 2050 [1]. The rate of production of new antibiotics is declining [2] and the UN states that resistance to antibiotics is a global health threat requiring urgent attention [3, 4].

A contributing factor to antibiotic resistance is the misuse of antibiotics in hospitals [5–7]. A recent study in Malawi found that one-third of bacteria grown in blood cultures from hospitalized children were resistant to Ceftriaxone, the most commonly used parenteral antibiotic in the hospital [8]. Resistance additionally results in longer hospital stays for patients [9], increased costs [10] and death [11].

A rational use of antibiotics is necessary for optimizing patient outcomes [11]. Critically ill patients admitted to Intensive Care Units (ICUs) are at greater risk of serious morbidity and death if antibiotic therapy fails [11–13]. Malawi, a low-income country in Africa, has 25 ICU beds for a population of 17 million people [14, 15]. The current use of antibiotics in ICUs in Malawi is not well documented and there are no national guidelines for the use of antibiotics in ICUs. The aim of the study was to describe the use of antibiotics in a Malawian ICU.

## Methods

A retrospective review of medical records for all admissions to the main ICU in Queen Elizabeth Central Hospital in Blantyre, Malawi between January 2017 and April 2019.

### Study setting

Queen Elizabeth Central Hospital (QECH) is a large, state-run hospital in Blantyre, Malawi with 1000 beds. The hospital serves an immediate catchment population of 1 million people and is a referral center for the southern region of Malawi and the whole country. The main ICU has four beds and admits patients from all wards and all specialties in the hospital. The closed ICU is run by the department of anesthesia and intensive care and has continuous observation with a 1:1 nurse: patient ratio, vital signs monitoring, oxygen supplied by cylinders, electric suction, mechanical ventilation for the four beds, and can deliver vasoactive infusions with syringe drivers.

### Study population

All patients admitted to the ICU between January 2017 and April 2019. Patients who lacked data on prescribed and received medications were excluded.

### Data collection

Data for the study were extracted from the ICU patient register. The register contains information on clinical parameters at admission, diagnoses, demographics and treatments both prescribed and given, (some patients were prescribed a treatment by clinicians, but were not given the treatment due to stock-outs or other reasons), for all patients admitted to the ICU. Medication prescriptions in the ICU are primarily done by the admitting anesthetic clinical officer or anesthesiologist often in consultation with other specialty physicians. The specialty team the patients belongs to at times reviews prescriptions and can modify in agreement with the anaesthetist in the ICU. Admission data were extracted to a paper-based data collection tool from the patient observation charts by the research team from January 2017 to November 2017, and by the nurses and clinicians in the ICU from December 2017 onwards. Data on antibiotic treatments during the ICU period were added to the register from patient treatment charts and blood culture results were added to the register from the hospital’s laboratory database, which is run by the neighboring research affiliate, the Malawi-Liverpool-Wellcome Trust. Other types of cultures are not done in the hospital. The laboratory uses bioMérieux BACT/ALERT 3D for detecting microorganisms in blood. Routine standard laboratory procedures were followed by qualified laboratory personnel and antibiotic sensitivity profiles interpreted according to British Society for antimicrobial chemotherapy guidelines. Quality controls are performed using known Public Health of England National Collection of Type Cultures bacterial reference strains and the laboratory participates in external UK and USA based quality assurance. The data were entered into the electronic register database using double-data-entering and quality-checking by two staff members, and follow-up of missing data by the ICU clerk. An anonymous extract of the data was used for the study.

### Data analysis

Descriptive data were summarized using proportions, means, ranges, medians and interquartile ranges where appropriate. The many diverse diagnoses were grouped into eight categories, (serious infection, non-communicable disease [e.g. cancer, anaemia, unspecified tumours], trauma, bowel perforation or obstruction, post-delivery or abortion care, acute respiratory disease, pre-eclampsia/eclampsia, other/unknown), by the researchers. The number of antibiotics given to each patient was recorded, and the usage of more than one antibiotic was classed as concurrent or sequential. Concurrent use was a patient receiving two or more antibiotics at the same time. Sequential use was when the first antibiotic was discontinued and subsequently a second antibiotic was commenced. Blood cultures were classified as being taken on the first day of admission to ICU, taken during the ICU stay, taken during the peri-ICU period – from 5 days before ICU admission to 5 days after ICU discharge – or taken at any point during the hospital stay. Blood culture results were classified as positive or negative, and laboratory results stating, “no significant growth (contaminant)”, were classified as negative. Sensitivities to ceftriaxone of the cultured bacteria were noted. Data analysis was done with STATA (Release 15, StataCorp, College Station, TX). Ethical clearance was granted by the College of Medicine Research and Ethics Committee (COMREC P.07/18/2433)***.***

## Results

There was a total of 740 admissions to the ICU during the study period. Six hundred and forty patients had information on prescribed and received medications and were included in the analysis. Their median age was 32 (IQR 22–45) and 326 (51.0%) were male. The surgical and medical departments contributed more than half the total number of participants with 213 (33.3%) and 131 (20.5%) respectively. Post-operative admissions accounted for 352 (55.0%) of the participants. The participants’ median length of stay in ICU was 2 days (IQR 1–4), 34.8% died in-ICU and a total of 45.5% died in-hospital. (Table 1). The characteristics of the one hundred patients who were excluded due to missing information about medications did not differ substantially from the included participants (Supplementary Table 1).

**Table 1:** Patient and Infection characteristics

Variable	n (%) ***N*** = 640
Male sex	326 (51.0%)
Age in yrs. Median (IQR)	32 (22–45)
Specialty	
Surgery	213 (33.3%)
Medicine	131 (20.5%)
Obstetrics and Gynaecology	108 (16.9%)
Neurosurgery	105 (16.4%)
Peadiatrics	39 (6.1%)
Ear, Nose and Throat	19 (3.0%)
Other^a^	25 (4.0%)
Admitted from	
Theatre	352 (55.0%)
Emergency department	137 (21.4%)
Ward	128 (20.0%)
Recovery room	3 (0.5%)
Other	20 (3.1%)
**Diagnosis**	
Serious Infection^b^	141 (22.0%)
Non-communicable disease^c^	133 (20.8%)
Trauma^d^	103 (16.1%)
Bowel perforation or obstruction^e^	98 (15.3%)
Post-delivery or abortion care	43 (6.7%)
Acute respiratory disease^f^	35 (5.5%)
Pre-eclampsia/eclampsia	16 (2.5%)
Other/ Unknown	71 (11.1%)
Length of stay in days Median (IQR)	2 (1–4)
Duration of care with antibiotics in the ICU Median (IQR)	2 (1–4)
ICU mortality	223 (34.8%)
Length of stay for those who died Median (IQR)	2 (1–5)
Hospital Mortality	291 (45.5%)

^a^ Orthopedics, Burns and Oncology

^b^Including Meningitis, Malaria, Endometritis, Pneumonia, sepsis/septic shock and Tuberculosis

^c^ Cancer, Anaemia, unspecified tumours

^d^Including Head injury and Burns

^e^including Typhoid perforation

^f^ includes Asthma and Pulmonary Oedema

### Antibiotic usage in ICU

**Fig. 1:**
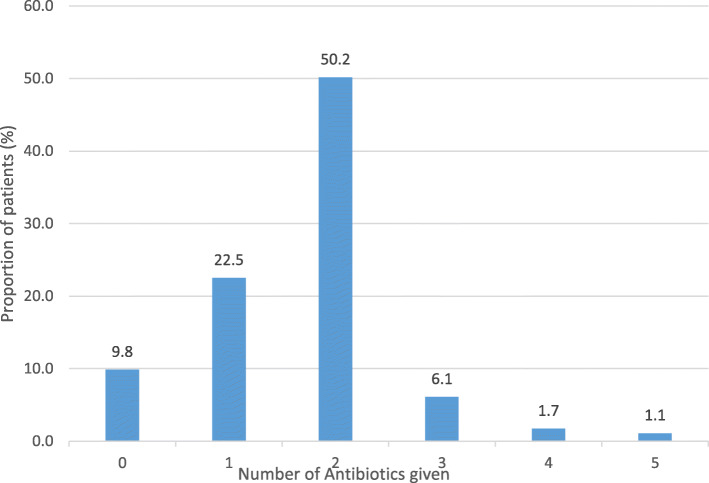
Number of antibiotics given to the patients in ICU

Of the 640 patients, 522 (81.6%) were given an antibiotic. The modal number of antibiotics received was two and 18 (2.8%) received four or five different antibiotics during their stay in ICU (Fig. 1). In total, 577 patients (90.2%) were prescribed an antibiotic and fifty-five (9.5%) of the patients were prescribed but did not receive the antibiotic.

The most commonly used antibiotic was ceftriaxone, given to 470 (73.4%) of the patients. The standard dose of ceftriaxone to patients with normal renal function was 2 g, given once a day - the frequency increased to twice a day in patients with meningitis. Metronidazole was given to 354 (55.3%) and meropenem to 30 (4.7%) patients (Table 2). Three-hundred-and-thirty-three (52.0%) of the participants received more than one type of antibiotic concurrently – ceftriaxone and metronidazole was the most common combination, given to 317 patients (49.5%), 58 patients (9.1%) received a different combination. Forty five patients (7.0%) were given different antibiotics sequentially.

**Table 2:** Antibiotics prescribed and given in the ICU

Antibiotic	Number of courses prescribed ***N*** = 1024	Number of courses given ***N*** = 933	Proportion of all antibiotic courses given ***N*** = 933	Proportion of patients who were given the antibiotic ***N*** = 640
Ceftriaxone	520	470	50.4%	73.4%
Metronidazole	378	354	38.0%	55.3%
Meropenem	43	30	3.2%	4.7%
Ciprofloxacin	17	17	1.8%	2.6%
Cotrimoxazole	15	12	1.3%	1.9%
Piperacillin Tazobactam	13	13	1.4%	2.0%
Gentamycin	13	13	1.4%	2.0%
Doxycycline	4	4	0.4%	0.6%
Amoxicillin Clavulanate	3	3	0.3%	0.5%
Benzyl penicillin	3	3	0.3%	0.5%
Azithromycin	3	3	0.3%	0.5%
Flucoxacillin	2	2	0.2%	0.3%
Amikacin	2	2	0.2%	0.3%
Erythromycin	2	2	0.2%	0.3%
Amoxicillin	1	0	0.0%	0.0%
Ampicillin	1	1	0.1%	0.2%
Ceftazidime	1	1	0.1%	0.2%
Chloramphenicol	1	1	0.1%	0.2%
Clindamycin	1	1	0.1%	0.2%
Cloxacillin	1	1	0.1%	0.2%

### Blood cultures

In total, 192 (30.0% of the patients) had a blood culture done during their stay in-hospital. One-hundred-and-thirty seven- (21.4% of the patients) had a blood culture done in the peri-ICU period, of these 70 were done in the ICU (11.0% of the patients) and 9 (12.9% of the ICU cultures) were done in ICU on the day of admission. Of the eleven patients with positive ICU cultures, nine were treated with ceftriaxone. In five of these, bacteria were grown that were resistant to ceftriaxone. All the five patients received ceftriaxone before the blood culture was taken and no patient received ceftriaxone after resistance was found.

Of the 137 peri-ICU cultures done, 25 (18.3%) were positive, twenty three (16.8%) had a missing result, and 89 (65.0%) were negative. Of the 70 blood cultures taken in ICU, 11 (15.7%) tested positive. Among the patients who had a blood culture done in the peri-ICU period, 43.8% died while among those who did not have a blood culture done, 32.4% died.

Twenty-nine bacteria were grown in the 25 positive peri-ICU blood cultures and can be seen in Table 3. Five bacteria (17.2%) were sensitive to ceftriaxone, (three of the *Staphylococcus aureus*, one of the *Escherichia coli* and the *Salmonella typhi)*. Eight were not tested, and the remaining 16 were resistant to ceftriaxone, (five of the *Klebsiella pnemoniae*, five of the *Acinetobacter baumanii,* three of the *Escherichia coli*, two of *Alpha-haemolytic streptococcus,* and one of the *Proteus mirabilis).*

**Table 3:** Bacteria grown in the cultures

Bacteria	Bacteria grown in the Peri-ICU positive cultures *N* = 29	ICU cultures with positive growth of bacteria *N* = 13
*Klebsiella pnemoniae*	5	2
*Acinetobacter baumanii*	5	4
*Escherichia coli*	4	0
*Alpha-haemolytic streptococcus*	4	2
*Enterococcus faecalis*	3	1
*Group D streptococcus*	2	2
*Staphylococcus aureus*	2	0
*Proteus mirabilis*	1	0
*Salmonella typhi*	1	0
*Streptococcus pyogenes*	1	0
*Pseudomonas aeruginosa*	1	1

## Discussion

Among a population of patients in an ICU in Malawi, 81.6% received antibiotics during their ICU stay and more than half received two or more antibiotics. An additional 9.5% of patients were prescribed but did not receive an antibiotic – possibly due to medication stock-outs.

A frequent use of antibiotics in ICUs has been reported elsewhere [11, 16, 17]. In South Africa and Ghana, 75 and 71% of ICU patients were given antibiotics respectively [17, 18]. In our study we found ceftriaxone was the most commonly used antibiotic. This use of ceftriaxone is likely to be guideline triggered as the Malawi standard treatment guidelines (supplementary file 1) recommends that ceftriaxone be given as the initial course before a blood culture is done [19]. Cephalosporins and other broad spectrum antibiotics are also commonly used in ICUs in other countries [16, 20].

We have found a substantial use of more than one antibiotic concurrently. Some of this is likely to be rational – for example the most common combination was ceftriaxone and metronidazole which, with their differing spectrum of action are a rational choice for diseases such as abdominal sepsis. Irrational concurrent use may also have been prevalent but is difficult to judge due to the lack of available detailed clinical information. Irrational use of antibiotics has been reported to be a contributing factor to poor outcomes such as prolonged length of stay and death in ICU [21] and increases costs [22]. Indeed, efforts through antibiotic stewardship programs are being put in place to help combat the problem of irrational antibiotic use in healthcare both in Malawi and other countries [13, 23–26]. During this study period, there was no evidence of such programs in the ICU and we are not aware of programs tackling antibiotic use specifically in ICUs in Malawi.

While antibiotics in ICU are used for many types and sources of infections, blood cultures may be used to direct the appropriate choice of antibiotic [11, 27, 28]. Our findings of infrequent testing of blood cultures, the low yield of positive results and the predominant use of broad-spectrum antibiotics suggest that most antibiotics were being used empirically for patients with suspected infection, without the guidance of blood cultures. Despite the sparse positive blood culture results, we have found antibiotic resistance in the ICU. These findings are similar to other studies done in other ICUs in Africa [13, 29]. Resistance to ceftriaxone poses a major threat to the critically ill patients as other potent antibiotics for critically ill patients can be scarce in Malawi. The sparse use of blood cultures in the ICU, could be attributed to cost concerns, a lack of specimen collection bottles in the unit, a lack of awareness among clinicians, the fact that antibiotics are often initiated before admission to ICU so cultures may be seen as less clinically relevant, or poor communication with referring units about previous cultures and the need for repeat tests in the ICU.

The higher mortality seen in patients who had blood cultures taken may seem surprising. However, it is likely explained by local practices of taking blood cultures in more severely unwell patients, or in those who are not responding to treatment.

Our study is the first of its kind to look at the antibiotic usage in an ICU in Malawi, and links over 2 years of data from the ICU register to the laboratory database. Limitations of our single-center study include the lack of available medication data for some ICU patients and the limited available clinical information to understand the rationality of the antibiotic usage.

A structured antibiotic stewardship program focusing on what is feasible may be useful in ICUs in Malawi and other low-resource settings. Patterns of infectious diseases in the unit, current treatments and outcomes for the patients should be mapped out. Local susceptibility patterns should be studied and used to develop guidelines for initial antibiotic therapy for patients with suspected bacterial infections depending on diagnosis and clinical condition. Patients’ previous investigations, blood culture results and antibiotic treatments should be communicated between hospital departments. Where possible, blood cultures should be done on arrival to ICU, when a bacterial infection is suspected, and when a patient is not responding to treatment and should include sensitivity testing. Antibiotic therapy should be reviewed daily, and clinical and laboratory information used for decisions about whether to continue, modify, or discontinue therapy. Multidisciplinary teams should be involved in decisions, including ICU clinicians, nurses, specialty doctors and microbiologists [26].

## Conclusion

We have found a substantial usage of antibiotics in an Intensive Care Unit in Malawi. Ceftriaxone, the last-line antibiotic in the national treatment guidelines, is commonly used, and bacteria appear to show high levels of resistance to it, although blood culture testing is infrequently used. Structured antibiotic stewardship program may be useful in all ICUs.

## Supplementary information

Additional file 1: **Supplementary Table 1.** Characteristics of patients who were excluded due to missing information about medications in ICU. (DOCX 12 kb)

Additional file 2: **Supplementary Table 2.** Diagnoses and outcomes of the 25 patients with positive blood cultures in ICU. (DOCX 17 kb)

Additional file 3. Summary of Malawi Standard Treatment Guidelines. (DOCX 13 kb)

## Data Availability

The data used in this manuscript is not readily available for public use. A request can be made to the department that own the data through the corresponding author on raphkazidule@gmail.com.

## Abbreviations

COMREC: College of Medicine Research and Ethics Committee
ICU: Intensive Care Unit
IQR: Interquartile Range
QECH: Queen Elizabeth Central Hospital
WHO: World Health Organisation
