# Supplementary material for: The use of antibiotics in the intensive care unit of a tertiary hospital in Malawi
Source: BMC Infect Dis. 2020 Oct 19;20:776. doi: 10.1186/s12879-020-05505-6 (PMC7574463; doi:10.1186/s12879-020-05505-6)
Supplement: Supplementary file 1 — Supplementary Table 1. Characteristics of patients who were excluded due to missing information about medications in ICU. (DOCX 12 kb) [file 12879_2020_5505_MOESM1_ESM.docx]

Supplementary table 1: Characteristics of patients who were excluded due to missing information about medications in ICU

| **Variable** | **n (%)**  **N=100** |
| --- | --- |
| Male sex | 49 (49.0 %) |
| Age in years. Median (IQR) | 29 (19-40) |
| Specialty  Surgery  Medicine  Neurosurgery  Obstetrics and Gynaecology  Peadiatrics  Ear, Nose and Throat  Other* | 20 (20.0%)  24 (24.0%)  23 (23.0%)  13 (13.0%)  10 (10.0%)  3 (3.0%)  7 (7.0%) |
| Admitted from  Theatre  Emergency department  Ward  Recovery room  Other | 42 (42.0%)  24 (24.0%)  23 (23.0%)  0  11 (11.0% |
| Diagnosis  Serious infection  Non-communicable disease  Trauma  Bowel Perforation or obstruction  Post Delivery or abortion care  Acute respiratory disease  Pre-eclampsia/eclampsia  Other/Unknown | 22 (22.2%)  30 (30.0%)  17 (17.0%)  7 (7.0%)  2 (2.0%)  2 (2.0%)  3 (3.0%)  17 (17.0%) |
| Length of stay in days Median (IQR) | 1 (0-2) |
| ICU Mortality | 34 (34.0%) |
| Hospital Mortality | 41 (41.0%) |
