# Supplementary material for: The use of antibiotics in the intensive care unit of a tertiary hospital in Malawi
Source: BMC Infect Dis. 2020 Oct 19;20:776. doi: 10.1186/s12879-020-05505-6 (PMC7574463; doi:10.1186/s12879-020-05505-6)
Supplement: Supplementary file 2 — Supplementary Table 2. Diagnoses and outcomes of the 25 patients with positive blood cultures in ICU. (DOCX 17 kb) [file 12879_2020_5505_MOESM2_ESM.docx]

**Supplementary Table 2 Diagnoses and outcomes of the 25 patients with positive blood cultures in ICU**

| Patient Serial number | Age category | Primary diagnosis | Other diagnoses | Infection site/source | Type of Bacteria grown | ICU outcome | Hospital Outcome |
| --- | --- | --- | --- | --- | --- | --- | --- |
| 1 | Child | Typhoid perforation | - | Abdomen | Staphylococcus aureus | Died | Died |
| 2 | Elderly | Pneumonia | Arthritis | Respiratory | Group A streptococcus | Died | Died |
| 3 | Child | Head Injury | - | Infected traumatic wound | Pseudomonas aeruginosa | Died | Died |
| 4 | Adult | Peritonitis | Bowel Perforation | Abdomen | Group D streptococcus  Klebsiella Pneumoniae | Died | Died |
| 5 | Adult | Sepsis | - | Abdomen | Enterococcus faecalis | Discharged | Discharged |
| 6 | Adult | Ruptured Uterus | - | Uterus | Group D streptococcus  Escherichia coli | Discharged | Discharged |
| 7 | Child | Bowel Obstruction | Sigmoid Volvulus | Abdomen | Enterococcus faecalis | Discharged | Discharged |
| 8 | Child | Pericardial Effusion | Sepsis | Unknown | Staphylococcus aureus | Discharged | Discharged |
| 9 | Child | Plexus Papilloma | - | Unknown | Klebsiella Pneumoniae | Died | Died |
| 10 | Adult | Cardiac Asthma | Hypertensive Crisis | Unknown | A-Haemolytic streptococcus | Died | Died |
| 11 | Adult | Bladder Injury | Septic Shock | Pelvis | Proteus Mirabilis | Discharged | Discharged |
| 12 | Adult | Bowel Perforation | - | Abdomen | Acinetobacter Baumanii  Enterococcus faecalis | Discharged | Discharged |
| 13 | Neonate | Bowel Obstruction | - | Abdomen | Klebsiella Pneumoniae | Died | Died |
| 14 | Adult | Anastomotic Leak | Cervical Cancer  Bowel Perforation | Abdomen | A-haemolytic streptococcus | Died | Died |
| 15 | Elderly | Bowel Perforation | - | Abdomen | Escherichia Coli | Died | Died |
| 16 | neonate | Tracheoesophageal Fistula | - | Oesophagus | Klebsiella pneumoniae | Discharged | Discharged |
| 17 | Adult | Pneumonia | Myocardial Infarction | Respiratory | Acinetobacter baumanii | Died | Died |
| 18 | Adult | Sepsis | - | Abdomen | Acinetobacter baumanii | Died | Died |
| 19 | Adult | Peritonitis | - | Abdomen | Klebsiella pnemoniae | Discharged | Discharged |
| 20 | Adult | Perforated appendix | - | Abdomen | Salmonella Typhi | Died | Died |
| 21 | Neonate | Sepsis | Gastro enteritis | Abdomen | Escherichia coli | Discharged | Discharged |
| 22 | Elderly | Faecal Peritonitis | - | Abdomen | Escherichia coli | Died | Died |
| 23 | Adult | Endomyometritis | Bowel Perforation | Abdomen | Acinetobacter baumanii | Discharged | Died |
| 24 | Adult | Eclampsia | - | Unknown | Acinetobacter baumanii | Discharged | Died |
| 25 | Adult | Pancytopenia | Right eye rupture  Pulmonary Oedema  Septicemia | Unknown | Staphylococcus aureus | Discharged | Discharged |

Neonate= less than 30 days old, Child below 18 years, adult 18 years to 50 years, elderly above 50 years.
