# Supplementary material for: The use of antibiotics in the intensive care unit of a tertiary hospital in Malawi
Source: BMC Infect Dis. 2020 Oct 19;20:776. doi: 10.1186/s12879-020-05505-6 (PMC7574463; doi:10.1186/s12879-020-05505-6)
Supplement: Supplementary file 3 — Summary of Malawi Standard Treatment Guidelines. (DOCX 13 kb) [file 12879_2020_5505_MOESM3_ESM.docx]

Summary of Malawi Standard Treatment Guidelines

The Malawi Standard treatment guideline aims to provide guidance on diagnosis and treatment of common health conditions found in Malawi. It incorporates, prescription guidelines, drug management as well as the Malawi Essential Medicines List. The guidelines is in its 5^th^ version which was published in 2015.

Disease conditions are grouped into organ systems e.g. cardiovascular conditions, Nature of illness e.g. infectious diseases and causative agent. e.g. Parasitic Infections.

Each disease conditions has a brief description of signs and symptoms a patient may present with, and treatment recommendations incorporating what medication the patient should be given first to the last line of medication if symptoms persist. For most bacterial infections the guidelines recommend empirical ceftriaxone, 2g intravenously, as first line treatment.

Medications are categorized into three categories.

**Level of use code** (H, D or C)

In this category, drugs are categorized for use according to the level of the health institution. H=Health Centres and above. D= District Hospitals and above. C= Central Hospitals only.

**Therapeutic priority code** (V or E)

This code identifies the therapeutic importance of each item.

**V (vital drugs).** These are drugs that are potentially life-saving, have significant withdrawal side-effects requiring regular supply and are or major public health importance (e.g needed by many patients for treatments of serious contagious diseases and needed to control epidemics)

**E (Essential) drugs which**: are effective against less severe, but nevertheless significant forms of illness.

**N (Non-essential)** drugs which are: used for minor self-limiting illness, of questionable efficacy, and have high cost for a marginal therapeutic advantage.

**Procurement System code (A or B)**

**A-List items-** Are required for large numbers of patients, will be routinely procured and stocked by central medical stores and include all H level drugs.

**B-List items-** Are generally required for limited number of patients, will not be routinely procured and stocked by central medical stores, payment for these must be made in advance prior to procurement by central medical stores.

Medications commonly used in the ICU e.g ceftriaxone, metronidazole and meropenem are categorized as Vital on therapeutic priority and A listed for the procurement code. Which means their availability is prioritised. While drugs like Piperacillin Tazobactam are categorized as essential drugs coded as effective against less severe but nevertheless significant forms of illness and B listed in the procurement system which means are not prioritised.
